# Hypocrisy is the tribute that vice pays to virtue: Dynamics of perceived organization hypocrisy and job embeddedness in the hospitality industry

**DOI:** 10.3389/fpsyg.2022.1036320

**Published:** 2023-01-13

**Authors:** Faheem Zeb, Qingjin Wang, Asad Shahjehan

**Affiliations:** ^1^Business School, Qingdao University, Qingdao, China; ^2^Department of Management Sciences, Hazara University, Mansehra, Pakistan

**Keywords:** organizational hypocrisy, job embeddedness, perceived inducement breach, future anxiety, COVID-19

## Abstract

In the wake of the COVID-19 pandemic, hospitality institutions are striving for legitimacy, which leads them to organizational hypocrisy, generating perceptions of inducement breach, future anxiety, and ultimately reduced Job Embeddedness. This study has identified industry and environmental situation-specific constructs in a mutual relationship to fill a theoretical gap. An electronic survey of 2100 frontline employees was administered among which 842 completed surveys were retained for analysis. The validity of the measures and the absence of common method bias were established. SPSS PROCESS was used to compute the serial mediation effects. Contrary to existing knowledge, the results of this study indicate that organizational hypocrisy increases employee job embeddedness. Three reasons identified for this result are Asian culture sample, prevalence of COVID-19 pandemic, and the necessity of hypocrisy emphasized by scholars. The study also presents an underlying mechanism that makes this relationship negative through perceived inducement breach and future anxiety. This study focuses on HOW and IF organizational hypocrisy has detrimental effects, thus adding empirical evidence to otherwise exploratory literature. For hospitality industry, employees are an irreplaceable resource that provides competitive advantages; they need to align their values with that of their employees by word and actions or risk losing them.

## Introduction

In the twenty-first century, exponential advances in the fields of science, technology, communications, politics, and sociology have forced organizations to adapt to these changes and developments. Especially, the current coronavirus 2019 (COVID-19) pandemic, caused by the novel severe acute respiratory syndrome coronavirus, has transformed how individuals and organizations interact with their internal and external environment. As governments have implemented numerous health measures, such as mandatory wearing of masks, social distancing, and quarantining, it has caused significant psychosocial and economic effects (Montemurro, 2020). Following suit, hotels and hospitality institutions must also incorporate these changes in their environment. According to the theory of “new institutionalism,” it is vital that organizations acquire and preserve legitimacy during their engagement with the external and internal environments as it is required to gain access to the services needed to sustain their existence (Meyer and Rowan, 1977; Nickell and Roberts, 2014). For maintaining legitimacy, managers of the hospitality industry respond to external challenges by implementing symbolic or ceremonial interventions and transitions in their internal structures (Brownell, 1990). However, in the hospitality industry, disparity between the rules and actual practices has been widely observed (Faldetta et al., 2013). It can even be stated that on paper, hospitality organizations may have a perfectly functional framework and procedures, yet it only exists on paper, as industry laws, guidelines, and procedures viewed as tools of legitimacy, pressurize the hospitality organizations to meet internal environmental demands. This discord between the actual practices of the organization and its illusionary display of managerial rationality, formalism, and intellectual rigor has led to incongruence. Brunsson (1989) has labeled this incongruence as “organizational hypocrisy” (OH) and described it as a loosely coupled connection between managerial statements, ideas, and words with actual institutional practices. In literature, several authors have highlighted this lack of consistency between the organization's anticipated legitimacy and their actions, as Orton and Weick (1990) state this as a loose coupling between industrial and regulatory systems and institutional everyday activities. Similarly, Meyer and Rowan (1977) suggest that institutions may devise laws and conventions for internal and external legitimacies without ever putting them into practice. These incongruences create a gap between formal systems established through irreconcilable pressures and uncertainties from the external environment and what actually is prevalent in the internal environment (Brunsson, 1989). Pérezts and Picard (2015) thus conclude that this gap between what is being stated to the external environment for legitimacy and the non-compliance to these rules, regulations, organizational framework, and structures often leads to OH.

Studies on OH are limited and non-existent in the hospitality industry. Furthermore, the existing studies are based on logic rather empirical research. Among the empirical studies, few studies have assessed the effects of OH on organizational stakeholders, especially the employees. Garrett (2017) identified OH as a situational factor that affects the ability of employees in displaying positive and negative attitudes and behaviors. Brunsson (1986) has identified that in order to achieve external legitimacy organizations could indulge in OH. He also proposes that this breeds mistrust and a sense of psychological contract breach (PCB) among the employees. This form of PCB that can be an outcome of OH is perceived inducement breach (PIB) as it is also the difference between the inducements committed by the employer and the inducements employee perceives that he/she has received. Moreover, there is consensus among the scholar that any form of PCB in general, and PIB in specific, leads to negative outcomes among employees. In this study, depending on the current situation of the COVID pandemic, it is proposed that employee anxiety about future potential events and the presumption about the harmful or negative events can be amplified due to the prevalence of perceptions of inducement breaches from the employers. Finally, in literature, there is also consensus that anxiety among the employees leads to negative consequences, and thus for this study, we propose that it would ultimately affect the Job embeddedness (JE) of the employees.

The purpose of this study, keeping in view the current external situation due to the COVID-19 pandemic, is to identify how hotels and hospitality institutions striving for legitimacy could lead them to OH. Their hypocritical practices, forced on them due to the external situations, could create a perception of inducement breach among the employees thus generating future anxiety (FA) and ultimately affecting their employees' JE. This study, for the first time, has identified the industry and environmental situation-specific forms of PCB, anxiety, and embeddedness that are PIB, FI, and JE in a mutual relationship to fill a gap in theory. Furthermore, this study also presents how OH harms the psychological, emotional, and behavioral outlook of frontline workers.

## Theoretical background and hypothesis development

### Organizational hypocrisy

To understand the definition of OH, it is important that we first comprehend the word hypocrite. The origin of the word hypocrisy is from the theater. According to Runciman (2009), the word hypocrite originates from the Greek term “hypokrisis,” which means “playing a part by pretending to be something one is not.” Furthermore, according to Fernando and Gross (2006), hypocrite is a person “who espouses higher standards than the real situation and pretends to use virtue, sacrifice, loyalty, commitment, idealism, and sympathetic concern for selfish ends.” In actuality, hypocrisy is the inability of an individual to practice what he/she preaches have inconsistent attitudes, originating from deceitful behavior (Hale and Pillow, 2015). In general, hypocrisy can be defined as inconsistency between three main things: talk, decisions, and action. The talk relates to organizational disclosure that includes informal agreements, group discussions, and interpersonal deliberations. Decisions include formal rules, regulations, policies, and written agreements recorded within the organizational hierarchy. While actions include what and how the organizational stakeholders perform and behave. OH can then be described as a voluntary behavior that does not comply with the values proclaimed by the organization and the associated expectations; the contradiction between the organization's advocated theory and the theory in practice; a difference between encouraged and applied fundamental beliefs, value; and finally stating and propagating that organization uses a single norm system, while in actuality practicing a multiple norm system. Specifically, OH relates to inconsistencies or disjuncture during informal agreements formed verbally, formal decision-making resulting from formal discussions and deliberations, and actions performed by the organization in contradiction of what was formally or informally agreed upon. Brunsson (1989) relates to OH as “a fundamental type of behavior in the political organization: to talk in a way that satisfies one demand, to decide in a way that satisfies another, and to supply products in a way that satisfies a third” (p. 27). Although organizations can tactically employ inconsistencies in their talk, decisions, or actions in order to accomplish their goals however these hypocritical practices are reflected in the environment and their gains are short-lived. According to Meyer and Jepperson (2000), organizations are inherently hypocritical since they face overlapping outcomes and behavior endowed with social agency.

Organizations and administrators are required to “walk their talk,” or at the very least try to follow what they preach. According to (Weick, 1995), “walk the talk” approach provides an appropriate buffer against hypocrisy. Although acting hypocritically bridges the gap between the organizational image and its daily functionalities, yet this also leads to a loss of organizational credibility (Christensen et al., 2010). More specifically contradiction between language and action is referred to as hypocrisy, has inconsistencies in their external institutional and normative demands (Lipson, 2006), and is incorporated into their inner structures. OH is often caused by inept reactions to competing for external pressures by loosely coupled or decoupled organizational elements within the organizations (Weick, 1995), and these contradictory organizational environment pressures are ultimately reflected in their structures, processes, and philosophies (Brunsson, 1986). If there is a disagreement between divergent parties on certain issues, various types of ideologies can emerge in organizations. As a result, such political organizations foster distrust and cynicism within their inner structure. Furthermore, OH can foster a feeling of PCB among members and observers and in turn a fear about their future. A cynical attitude like FA may lead to a widespread lack of affiliation and embeddedness toward the organization. As Foote (2001) clearly states that “The gap between rhetoric and reality may also erode job security” therefore, once the level of hypocrisy becomes excessive, it is believed to breed pathological effects in organizations, such as a lack of embeddedness from its members.

### Perceived inducement breach

Due to the exceedingly competitive industry and rapid technological advancements, a significant corporate transformation has occurred, resulting in changes in employment relationships among staff at all levels (Buhalis et al., 2019; El Hajal and Rowson, 2020). These developments have triggered a significant amount of scholarly interest, especially in the study of employees' reactions to various forms of work relationships (Chan and Jepsen, 2011; Shehawy, 2022) and psychological contracts (Petery et al., 2021). The psychological contract is defined by Rousseau (1998) as “the mutual expectations held by employees and their employers regarding the terms and conditions of the exchange relationship.” In an employment environment, a psychological contract is between two parties that provide an enduring mental model of the employment relationship, presents a comprehensive understanding of what to expect from one another, and directs the parties toward effective interactions (Rousseau, 2004). In the literature regarding the psychological contract, a large portion is focused on addressing the PCB, which occurs when one party in a relationship believes the other party has failed to fulfill promised obligations (for example, Robinson and Rousseau, 1994; Morrison and Robinson, 1997). Generally, research has focused on the PCB from the employees' perspective and how their perceptions regarding the breach affect their commitment toward the organizations, their ability to display organizational citizenship behavior, satisfaction from job and organizations, task and non-task performance, and most importantly for the study in context their level of embeddedness toward their current job (Coyle-Shapiro, 2002; Kickul et al., 2004; Raja et al., 2004; Rousseau, 2004). By employing PIB (Chen et al., 2008), this current study strives to explore the question of “How employees respond to OH that leads to a perception of inducement breach?”

With psychological contracts, the core issue is of “the belief that a promise has been made and a considerations offered in exchange for it, binding the parties to some set of reciprocal obligations” (Rousseau, 1989), while obligation in this definition is a willingness to do something in the future. When employees enter into an employment contract, they understand that by doing so, they accept an obligation to provide specific services to the organization and to follow management's directives. In addition, the employee also believes that the organization is obligated to include certain incentives in return for the employee's efforts, such as pay, benefits, training, and job prospects. The concept of inducements was first presented by Barnard (1968) and further explained by March and Simon (1958) state that “the employer is obligated to provide a set of inducements in exchange for the employee's obligations to provide certain contributions.” The obligations created are perceived as mutual in nature, as they are created through written, oral discourse, or even actions performed by either employer or employees. Both sides believe that their responsibilities are mutually acknowledged, although communication is frequently inadequate or inconsistent. These miscommunications could be interpreted as deliberate or harmful by the employee leading to the perception of too many promises made than were actually intended. Robinson and Rousseau (1994) state that this situation is in which the employee feels a PCB by the employers. Keeping into consideration the shaped perceptions of obligation, the current study analyzes how employees react to PIB by the employers based on the OH demonstrated in the organizations.

Based on the definition by Morrison and Robinson (1997) in this study, we defined PIB as the difference between the inducements committed by the employer and the inducements employee perceives that he/she has received. Studies on PIB present a negative relationship with organizational commitment, OCB, task and non-task performance, and more importantly, with JE. These studies also present a positive relationship with cynicism and FA. In addition, social exchange theory (Blau, 1964) and equity theory (Adams, 1965) both support these results. Both these theories posit that Employees pursue equal and equitable exchanges with their employers. Employees with psychological contracts breached by employers are more likely to feel that they cannot be relied on to meet their commitments and are unconcerned about employee wellbeing (Robinson and Bennett, 1995). As the balance of the exchange relations is disturbed, the employees start feeling anxiety at their job generally and FA in specific as they think if the employer has indulged in PIB in past, similar patterns could be expected from them in the future also. Similarly, there is sufficient research that states that PCB in general and PIB in specific increase employee turnover intentions and decrease JE.

### Future anxiety

Lewin (1942) concept of “life space” is usually employed in psychology to understand the concept of the future. According to the advocates of this theory (Pourbagher et al., 2014), the future consists of several dimensions like FA, future time perspective, and hope. Among these dimensions, FA could have antecedents like PCB, PIB, or OH. According to Zaleski (1996), FA refers to attitudes about the future wherein pessimistic emotional and cognitive mechanisms outweigh optimistic aspects and uncertainty outweighs optimism. It is the concern about future potential events and the presumption that harmful or negative events can arise. Each form of fear is related to the future; however, FA corresponds to a remote rather than immediate future. Furthermore, it is a personal preoccupation with, concern over, and fear of potentially negative developments that might occur in the future. It also has the ability to transform into hysteria or panic in serious cases. In comparison with other forms of anxiety, FA is cognitive rather than an emotional character as the individual seems to know about it and is aware of his/her situation. Zaleski (1996) further presents FA as a personality trait that determines how an individual responds to fears, personal experiences, and current happening in life. Authors like Eysenck and Calvo (1992) or Sorrentino et al. (1992) also support this understanding of FA. Eysenck and Calvo (1992) explain it through the theory of hyper vigilance and posits that early identification of threat signals is a core feature of FA. Whereas, Sorrentino et al. (1992) explain this concept through the concept of uncertainty orientation which is a tendency of some individuals to perceive life as an unknown.

In organizational psychology there exist similar concepts to FA such as general anxiety, fear, future time perspective, and worry; however, there are major differentiations among them. Anxiety is described as a threat feeling that is unpleasant and unspecified, experienced subjectively in the form of negative tension, and is linked with physiological changes (Eysenck, 2000). In contrast, FA is a form of anxiety that relates to the future in general. The difference between fear and FA is rather distinct, as fear is caused by known cues (Lewis et al., 2010) and results in self-preservation behaviors, such as escape and avoidance (Lang et al., 1997). In literature, different forms of anxiety are distinguished by the emotional position an individual assumes regarding an undesired situation in the near or distant future. Another concept that is most closely related is “Future time perspective.” It is a relatively stable tendency of an individual to focus on his/her future by thinking about it, planning for it, and setting goals and objectives to reach it (Zimbardo and Boyd, 2008). Carelli et al. (2011) present the future time perspective as positive which focused on the positively evaluated future, or negative which relates to the negatively evaluated future. FA most closely relates to a negative future time perspective, or as stated by Zaleski (1996), its most basic and important element along with other elements like fear of the future, anger, aversion, and feeling of helplessness (see Zimbardo and Boyd, 2008; Carelli et al., 2011). Worry is another similar construct, however, its relationship with FA is dependent upon how we define it. Boehnke et al. (1998) define worry “as cognition that an object (self, in-group, society, world) will diverge from its desired state.” And conceptualized worry as a sub-construct of anxiety. Schwartz and Melech (2000) are of the view that worry and FA coexist only when the individual believes that transforming the current and expected states will be difficult. In contrast, if the individual has problem-solving skills, it is not necessary that worry would accompany FA. Our interest in FA, rather than other forms of anxiety, stems from the current situation of the COVID-19 pandemic and the social and political processes and events associated with it. Furthermore, in the current environmental situations, there exist constant open and latent organizational and job threats to the employees, which have visible consequences for the employees. In addition, the health problems and social alienation due to the current situation and its effects on the employees have also occupied our attention. Based on these scenarios, the question arises that how strong is FA among the employees of the hospitality industry? And how it affects the employees to rethink their relationship with their organization and job?

### Job embeddedness

Management literature has always strived to answer the question of why people leave and the probable answer to this question has been “they did not like their job” or “they have someplace else to go.” In contrast, the management scholars seldom ask the question as to why people stay, and the answer may well be the opposite of the reasons for turnover that is they liked their jobs or did not have any place else to go. Traditionally literature has identified affect-saturated constructs such as job satisfaction, organizational commitment, and job involvement in explaining turnover (Holtom et al., 2006). However, Mitchell et al. (2001) posits that in contrast to turnover, contextual influence most likely affects staying and JE. They have presented JE as “a broad constellation of influences on employee retention.” The experiments of “embedded figures” and “Field theory” could help in explaining the construct of JE (Lewin, 1942). The embedded figure tests are used to assess the concept of “field dependence—independence” and include a set of images that are immersed in their background and had to be separated from them. Good performance in this test shows field independence which is the capability to extract information from its surrounding gestalt or context. Similarly, field theory also presents the same concept which states that people have a perceptual life space that represents and links different facets of their lives. These links can be few or many and close or distant. Using these theories, we may define JE as a net or a web in which an employee is trapped. An employee who is highly embedded has a lot of close-knit, undifferentiated ties within the organization. The content of the parts can vary significantly, implying that a person can become entangled and embedded with the system in a number of ways. Thus, when describing embeddedness, the focus is on its overall level rather than its specific elements. Literature has identified three main aspects of JE labeled as “Links,” “Fit,” And “Sacrifice.” The link aspect of JE is referred to as the degree to which employees are connected to their peers or organizational activities. Second, the fit aspect is the degree to which one's job and organizational culture are analogous to or fit with other facets of their life spaces. Sacrifice is the last aspect of JE which is the level of easiness with which these connections can be severed. The three aspects link, fit, and sacrifice, and their interactions at the organizational environment and communal level provide a three-by-two matrix resulting in six dimensions that could explain JE. In summary, JE is the extent of an employee's “stuckness” or enmeshing, within a larger social system, and it results from numerous external (or contextual) forces—which are labeled links, fit, and sacrifice—in the organization and community that operate on a focal employee.

According to social exchange theory (Blau, 1964), there might be some instances when workers believe that their employer does not value employee contributions and is unconcerned about their wellbeing (Eisenberger et al., 2001). The norm of reciprocity is central to this concept as it forces the organizations to reward or respond positively to the employees that perform favorably (Gouldner, 1960). The theory further assumes that employee desire to remain embedded with the organization is damaged by the impression that greater efforts toward achieving institutional goals and objectives are not being rewarded. This situation is most closely related to OH because it is also a voluntary organizational behavior in which the stated values do not conform with the employees' expectations. A lack of committed workplace incentives, opportunities for growth and advancement, or desirable work conditions contribute toward OH even more, when the employees perceive that these hypocritic actions are voluntary (Eisenberger et al., 2001), thus resulting in a lack of obligation on employees to “repay” the organization. One form of repayment to the organization is to remain embedded in it and the display OH thereby deteriorates this bond or attachment. Based on the above discussion, it is reasonable to propose that OH negatively affects JE. Employees who feel let down by their organizations are more likely to show negative exchange relationships and display a lack of JE, thus weakening their links and ties with the organizations. Therefore, it is hypothesized for this study,

H1: Organizational hypocrisy negatively effects Job Embeddedness.

Based on the available literature, association between OH and JE may not be direct. Organizations indulging in OH breeds mistrust and a sense of PCB among the employees. It is proposed that PIB is a form of PCB that could be an outcome of OH, as it is the difference between the inducements committed by the employer and the inducements employee perceives that he/she has received. There are ample studies in which PCB, or its different forms, mediate the relationships between organizational factors (for example fairness, organizational justice, perceived politics) and employee outcomes (such as task performance, voice behaviors, turnover intentions, OCB, and CWB). In addition according to the principles of social exchange theory, an organization that fails to meet its obligations reduces workers' feelings to stay embedded and this minimal need to reciprocate due to a perception of inducement breaches could result in the reduction of their tenures. This leads us to the hypothesis that:

H2: PIB will mediate the relationship between OH and JE.

According to Killgore et al. (2021), individuals respond differently to a specific or an amalgamation of distressful situations. The magnitude of the COVID-19 pandemic has numerous negative consequences that could affect the employee–organization relationships (Liu et al., 2020; Melegari et al., 2021; Kayis et al., 2022). Research shows that historically the effect of significant outbreaks like natural disasters, terrorist attacks, and pandemics has negative psychological and psychosocial effect that may last for years after the incident (Bonanno and Mancini, 2008). One of its consequence is individuals reverting to future negative thinking which undermine their wellbeing (Holman and Silver, 2005). This phenomenon is observed in the current COVID-19 pandemic as individual worries about their present and future scenarios have increased (Usher et al., 2020; Melegari et al., 2021). Economic and social problems like economic collapse, risk of infection, and unemployment originating due to the pandemic may lead to anticipatory fear and concern about unfavorable scenarios in the future. Thus, we expect that the perceived threat of COVID-19 will increase FA. Both organizations and individuals are facing this pandemic and stirring to cope with its negative effects. As mentioned earlier, organizations always strive to acquire and preserve their internal and external legitimacy. It becomes vital for an organization in the current pandemic, as legitimacy is required to gain access to the services needed to sustain their existence. In pursual of legitimacy in these challenging times, organizations pursue both positive and negative practices for survival. In the hospitality industry, disparity between the rules and actual practices has been widely observed before the pandemic and is expected to have increased during it. Thus, it is proposed for this study that FA would be further augmented due to the current pandemic situation, if the employee perceives those inducements committed by the organization have not been fulfilled. Further due to the contextual nature (Mitchell et al., 2001) of employee FA, we can expect a significant influence on JE. Thus, the following hypotheses are proposed for this study.

H3: FA significantly mediates the relationship between OH and JE.
H4: There will be a significant serial mediation between OH and JE *via* PIB and FA.

**Figure 1:**
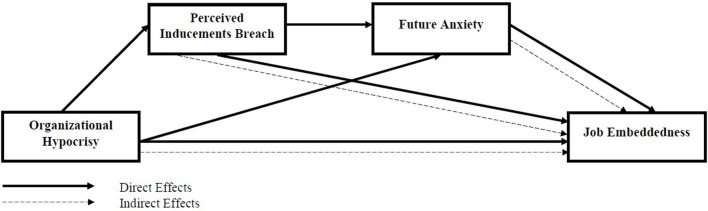
Model of the study.

Based on the theory development the model proposed for the study is shown in Figure 1.

## Materials and methods

### Sample and procedure

To test the theoretical framework and hypotheses, a team of 33 students from a post-graduate class was trained online to administer the survey instrument. Due to the COVID pandemic, it was strictly directed that the research team would not have any face-to-face contact with the respondents. The contact information of the respondents was extracted from a national business and organizational database. The research team, through an email, contacted twenty-one hundred employees from the hospitality industry to participate in an electronic survey. Several reminders for participation in the survey were sent to improve the response rate. In total, 936 (44.57%) surveys were returned, which were assessed for missing values and outliers, and finally, 842 (net response rate = 40.1%) completed surveys were retained for final analysis. Table 1 shows the descriptive characteristics of the sample.

**Table 1:** Demographics of the sample.

Gender	Male	593 (70.4%)
	Female	249 (29.6%)
Age in years	31.97 years (SD = 7.74)
Education in years	15.32 years (SD = 2.15)
Experience in years	7.60 years (SD = 6.41)
Supervisory status	Supervisor	361 (42.9%)
	Non-supervisory	481 (57.1%)

### Ethical consideration

The study was approved by the Departmental ethical review committee and was performed in accordance with the Declaration of Helsinki. To protect the participants' human rights, permission to collect data was granted from the head of the organization. Before the survey was administered, the purpose and process of the study, a guarantee of confidentiality, voluntary participation, anonymity of data and their right to discontinue participation at any time during the process were explained to the organizational heads and potential participants. The researchers explained to the participators the details of the concept and about the survey questionnaires, who decided to join in the survey and completed a written consent form. For the protection of the participants' personal information, the survey did not ask about information related to personal identity, and collected data were discarded when the study was finished.

### Measures

The participants completed the online surveys on organizational hypocrisy, perceived inducement breach, future anxiety, and job embeddedness. The description of the measure employed for each variable is as follows:

#### Organizational hypocrisy

A 17-item scale developed by Kiliçoglu et al. (2019) was used to measure organizational hypocrisy. The measure included three dimensions *Keeping Words into Practice* having 5 items such as “The administration embodies decisions taken by them.” The second dimension was *Compliance Between Internal Structure and The Environment* having eight items for example “Our organization reflects the environment's norms.” The third dimension *Inconsistency in Practices* included 5 items like “Although management says that they will solve problems in the organization, they don't.” The overall Cronbach alpha reported for the full scale is 0.728. All the items were scored on a 5-point scale ranging from 1 strongly disagree to 5 strongly agree.

#### Perceived inducement breach

A nine-item scale originally developed by De Vos et al. (2003) and Tekleab and Taylor (2003), and further adopted and refined by Chen et al. (2008) was used to assess perceived inducement breach (Sample item: In my job, I can make decisions by myself). A five-point too little/too much (TLTM) scale ranging from −2 “receive much less than promised” to +2 “receive much more than promised” was used to rate the items. According to Vergauwe et al. (2017), the TLTM scale provides additional incremental validity over traditional Likert scales. The scale's alpha coefficient for this sample is reported at 0.75.

#### Future anxiety

To measure future anxiety a 5-item scale developed by Zaleski et al. (2019) was employed for this study. All the items were rated on a five-point Likert scale ranging from 1 being False and 5 being true. The Cronbach alpha value of the scale for this sample was 0.81.

#### Job embeddedness

The sample self-assessed their job embeddedness through a three-dimensional measure developed by Crossley et al. (2007). The respondents were asked that “After considering both work-related (such as relationships, fit with job, benefits) and non-work-related factors (such as neighbors, hobbies, community perks)” (Karatepe and Avci, 2019) respond to the following three items “I am too caught up in this organization to leave,” “I feel tied to this organization,” and “I am tightly connected to this organization” on a 5-point scale ranging from 1 = strongly disagree to 5 = strongly agree. The Cronbach alpha value of the scale for this sample was 0.788.

## Results

### Confirmatory factor analysis

A confirmatory factor analysis (CFA) was conducted to test for the construct validity. The Model fit indices for the measurement model and alternate models were calculated, and the results of the four factor model (Organizational Hypocrisy, Perceived Inducement Breach, Future Anxiety and Job Embeddedness) presented the most suitable fit with the model, as it reported comparative fit index (CFI) = 0.914, non-normed fit index (NNFI) = 0.90, root mean square error of approximation (RMSEA) = 0.042, and root mean square residual (RMSR) = 0.04 consistent with Bagozzi et al. (1991) thresholds while the results of the alternate models did not adhere to these thresholds.

### Reliability and common method bias

We assessed the reliabilities of our scales, which could be observed in Table 2 that all are >0.70. Second, the average variance explained (AVE) for each variable was >0.5 and composite reliability (CR) is greater than the 0.07 threshold (Hair et al., 2010). Most importantly, the square root of AVE (the smallest being FA =0.708) is greater than all the correlations reported in Table 2 between the constructs of the study, thus providing support for the discriminate validity of our study measures. The multicollinearity diagnostics were also executed, and the variance inflation factors (VIF) were calculated for the explanatory variable. It was observed that all the VIF values were well below 10 (with maximum VIF reported = 1.248). Lastly, the multicollinearity diagnostics also reported variance proportions for all the variables which were assessed using the guidelines from Hair et al. (2010), and the highest condition indices were found to be lower than 0.6. Based on the aforementioned diagnostics, it could be concluded that for the interpretation of the study data, multicollinearity is not a major concern.

**Table 2:** Correlations and descriptives.

	**AVE**	**CR**	**Mean**	**SD**	**Gender**	**1**	**2**	**3**	**4**	**5**	**6**	**7**	**8**
Age			31.97	7.74	−0.07*								
Education			31.97	2.15	−0.03	0.03							
Experience			7.60	6.41	−0.02	0.76**	−0.08*						
Sup status			–	–	−0.02	−0.25	−0.15**	−0.25**					
PIB	0.507	0.902	3.45	0.58	−0.07	0.19**	−0.067*	0.13**	−0.09*	(0.75)			
FA	0.501	0.833	3.1	0.78	0.12**	−0.03	0.1**	0.013	−0.08*	−0.02	(0.81)		
OH	0.579	0.959	3.34	0.39	0.05	0.18**	−0.01	0.20**	−0.09*	0.35**	0.27**	(0.73)	
JE	0.655	0.849	3.17	0.83	0.07	0.13**	−0.06	0.20**	0.06	0.052	−0.23**	0.14**	(0.79)

** *p* < 0.01 level,

* *p* < 0.05 level (Cronbach Alpha).

As the data collected for this study was perceptual, it was required to assess whether common method bias augments relationships between the study variables. Initially, Harman's single-factor test was performed to assess potential common method bias. By employing exploratory factor analysis, six factors were generated having eigenvalues >1, and the first factor didn't explain majority of the variance (only 16.4%). In addition, with *p* < 0.01, the hypothesis of one general factor underlying the relationships was rejected. As Podsakoff (2003) has highlighted, several limitations of single-factor tests additional assessments were also conducted.

The model fit indices are also consistent with Bagozzi et al. (1991) thresholds which also further proof that common method bias is not a potential problem. Furthermore, according to Lindell and Brandt (2000), the smallest observed correlation among the model variables can function as a proxy for common method bias. In Table 2, it can be observed that the smallest correlation among the core study variables is −0.02 (between PIB and FA) thus providing additional evidence regarding the absence of potential common method bias.

### Descriptive statistics

First, the correlations between the study variables were assessed. The study has five control variables and some of their relationships with the key constructs have been found significant as shown in Table 2 (include the Means and SD of all variables and update the correlation table).

Gender had a significant positive relationship with FA, as 1 was coded for males and 2 for females, it can be inferred that females are more prone to FA than males (r = 0.12, *p* < 0.01). Age had a significant positive relationship with PIB (r = 0.19, *p* < 0.01), OH (r = 0.18, *p* < 0.01), and JE (r = 0.13, *p* < 0.01) which shows that an increase in age increases JE; however, it also increases the perception of an employee regarding the prevalence of OH and PIB at the workplace. The level of education has a significant negative association with PIB (r = −0.067, *p* < 0.05) and a significant positive association with FA (r = 0.1, *p* < 0.01). Thus, it is inferred that more educated employees develop more FA, yet they are also less sensitive to inducement breaches. Similar to age, experience also has a significant positive relationship with PIB (r = 0.13, *p* < 0.01), OH (r = 0.20, *p* < 0.01), and JE (r = 0.20, *p* < 0.01); thus, it could also be deduced from these results that increase in job experience increases JE and the perception that OH and PIB are prevalent in the organization. Lastly, the supervisory structure was coded as 1 for supervisors and 2 for non-supervisors; the results show that non-supervisory employees are less susceptible to PIB (r = −0.09, *p* < 0.05), FA (r = 0.08, *p* < 0.05), and OH (r = 0.09, *p* < 0.05). The correlation between the independent, dependent, and mediating variables presents interesting results. First, it was observed that PIB has a significant positive relationship with OH (r = 0.35, *p* < 0.01); however, it has non-significant relationships with FA (r = −0.02, NS) and JE (r = 0.052, NS). Expectantly, a significant positive relationship of OH (r = 0.27, *p* < 0.01) and a significant negative relationship of JE (r = −0.23, *p* < 0.01) with FA are reported. The most important result reported in Table 2 is the positive association between OH and JE (r = 0.4 *p* < 0.01), thus, reducing support for the H1 hypothesis of the study. Although this result is not aligned with the theoretical underpinning of this study, further verification of these results is presented in the succeeding analyses.

### Hypotheses testing

A serially mediated analysis was performed using regression analysis with the Statistical Package for the Social Sciences version 23. The SPSS PROCESS (Model 6) developed by Hayes (2012) was used to compute the mutual effects (10,000 bootstrap samples at 95% CI). This package provided a detailed insight into the direct and mediated effects of our study variables thus providing additional robustness to our findings. To establish the associations shown in Figure 1, the results of the serial-mediated regression analyses are presented in Table 3.

**Table 3:** Multiple regression analyses.

**Independent variables**	**Dependent variables**											
	**Model 1**		**Model 2**		**Model 3**		**Model 4**		**Model 5**		**Model 6**	
	**JE**		**JE**		**JE**		**JE**		**PIB**		**FA**	
	**β**	**t**	**β**	**t**	**β**	**t**	**β**	**t**	**β**	**t**	**β**	**t**
Gender	0.13**	2.17	0.12*	2.01	0.12*	2.01	0.17**	2.90	−0.09*	−2.32	0.16*	2.84
Age	0.00	−0.16	0.00	−0.33	0.00	−0.35	−0.01	−1.03	0.02**	3.94	−0.01	−2.33
Education	−0.01	−0.62	−0.01	−0.60	−0.01	−0.58	0.00	0.19	−0.02*	−2.68	0.03*	2.76
Experience	0.03**	4.33	0.03**	4.12	0.03**	4.11	0.03**	4.57	−0.01*	−2.18	0.01	1.01
Sup Status	0.18**	3.07	0.19**	3.19	0.19**	3.19	0.16**	2.74	−0.06	−1.45	−0.11	−1.98
OH			0.22**	2.97	0.21**	2.74	0.40**	5.10	0.51**	10.4	0.61**	8.53
PIB					0.01	0.16	−0.03	−0.64			0.13**	2.81
FA							−0.30**	−8.33				
R^2^	0.06**		0.07**		0.07**		0.14**		0.16**		0.11**	
ΔR^2^	0.06**		0.01**		0.00		0.07**		0.11**		0.08**	
F	9.93		9.82		8.41		16.65		26.16		15.02	

** *p* < 0.01 level,

* *p* < 0.05 level.

The regression analysis has presented some unexpected results; however, it also provided stronger support to the core study objectives. The control variables explain significant variance in JE (R^2^ = 0.06, *p* < 0.01) with gender (β = 0.13, *p* < 0.01), age (β = 0.03, *p* < 0.01), and supervisory status (β = 0.18, *p* < 0.01) having a significant positive effect on JE. To test the study hypothesis H1, OH along with the control variables was regressed against JE. The results of Model 2 show that OH has a significant positive effect (β = 0.22, *p* < 0.01) on JE. These results along with the positive correlation (r = 0.4, *p* < 0.01: Table 2) between these variables provide sufficient proof for the rejection of study hypothesis H1, which stated that “OH *negatively effects JE*.” Further examination of the occurrence of this result is conducted in supplementary data analysis.

To test the mediation-based hypotheses, direct effect of OH on JE was extracted from Table 3 and is shown in Table 4. The results are in line with the finding of Model 2 (Table 3) and a positive effect is reported (β = 0.39, *p* < 0.01). Based on the results in Table 4, indirect effects were calculated which are shown in Table 5.

**Table 4:** Direct effect of OH on JE.

	**Effect**	**SE**	**t**	***p***	**LLCI**	**ULCI**
OH	0.3968	0.0779	5.0962	< 0.01	0.244	0.5497

**Table 5:** Indirect effect(s) of OH on JE.

		**Effect**	**SE**	**LLCI**	**ULCI**
Model 1	OH = > PIB = > JE	−0.0162	0.0259	−0.0648	0.0387
Model 2	OH = > FA = > JE	−0.1839	0.0291	−0.2416	−0.1302
Model 3	OH = > PIB = > FA = > JE	0.0205	0.0078	0.0069	0.0374
	**Total indirect effects**	−0.1796	0.0398	−0.2498	−0.0975

Table 5 tests the three proposed mediation models of the study and also presents the overall indirect effects of the mediators. Model 1 in Table 5 provides statistics for testing the H2 hypothesis of the study which stated that PIB significantly mediates the relationship between OH and JE; however, the results show that the indirect effects of PIB though negative are not significant (β = −0.01, NS [LLCI = −0.0648, ULCI = 0.0387]). Thus, there is not sufficient support for the mediating effects of PIB. Model 2 (Table 5) tests the mediating effects of FA between the relationship of OH and JE. The results show that there are significant negative mediation effects of FA (β = −0.118, *p* < 0.01 [LLCI = −0.2416, ULCI = −0. 1302]). This provides support for the acceptance of study hypothesis H3.

**Figure 2:**
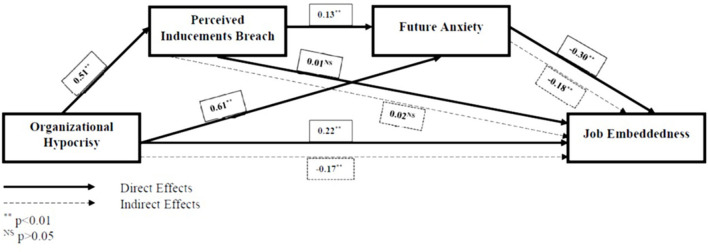
Model of the study with effect sizes.

Lastly, we have tested the overall model of our study and hypothesis H4. This was tested through serial mediation such that PIB was succeeded by FA between the independent and dependent variables. The results show that the serial mediation is significant (β = 0.02, *p* < 0.01 [LLCI = 0.0069, ULCI = 00.0374]) thus confirming the OH => PIB => FA => JE model proposed for this study. The total indirect effects of the model are negative and significant (β = −1,796, *p* < 0.01 [LLCI = −0.2498, ULCI = −0.0975]). The results are shown in Figure 2.

These results provide support for the core objective of this study, where OH does lead to negative consequences for the organization and the study at hand confirms that PIB and FA lead to a lack of JE through serial mediating effects. Furthermore, as OH has positive direct effects and PIB has insignificant direct (β = 0.01, NS) and indirect effects (β = −0.02, NS) on JE, the inclusion of a contextual variable FA modifies these relationships by turning the positive effects into negative and previously docile variables imperative.

### Supplementary data analysis

**Figure 3:**
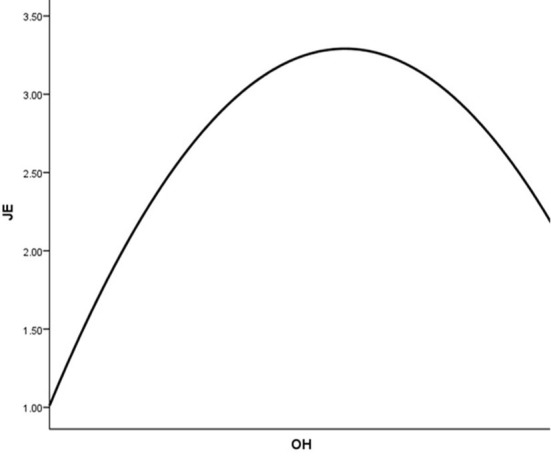
Curvilinear relationship between JE and OH.

One of the main results was not as proposed thus resulting in a lack of support for H1. Instead of a positive association between OH and JE, a negative correlation and effects were reported between them. For evaluating the dynamics of this result, we assessed the curvilinear relationship of OH and JE as according to Edwards and Berry (2010) in some cases the automatic assumption of linearity may hinder the precision of a theory. The curvilinear relationships are shown in Figure 3.

Figure 3 shows that JE has a curvilinear relationship with OH, and it is also significant as the quadratic function OH^2^ has β = −0.249 and *p* < 0.05. It could also be observed that the plot of the relationship is concaved into an almost n-shaped curve. This curvilinear plot partially explains our results: the prevalence of OH at a lower level could keep the employees embedded in their jobs; however, with an increase in OH, its positive effect weakens till the inception point beyond which it becomes negative. The figure also shows higher level OH could have negative effects on JE thus providing partial support to the theory. This supplementary analysis also shows that in this study sample the positive effect of OH is more powerful than its negative effect thus providing further support for the dismissal of hypothesis H1. In summary, the negative relationship between OH and JE could not be concluded in this study however keeping in view their curvilinear relationship the approval of this hypothesis could not be ignored for future studies.

## Discussion

This study hypothesized that employees' perceptions of OH decreased their ability to stay embedded. However, the results show the complete opposite of this proposition. We present three main reasons for the occurrence of this result contrary to the study hypothesis. First is the national culture of our sample. Pakistan is considered as a collectivistic society, characterized by a strong long-term commitment to the member group, whether it be a family, extended family, extended relationships, or a workplace. In a collectivist culture, loyalty is integral and transcends most other social norms and regulations, thus, creating strong bonds in which everyone accepts responsibility for their group members. Although scholars argue that in collectivist societies, offenses lead to humiliation and loss of credibility, and employer/employee relationships are viewed based on morality, this also leads to exploitation by decision-makers in these societies. Hypocritic actions and deeds are forgiven and ignored under the notion of loyalty and commitment to a group or organization. Effron et al. (2018) evaluated OH across 46 nations and their results show that managers that display more word-deed misalignment lack trust from their employees; however, the level of mistrust is higher in individualist cultures like North America and Western Europe than in the collectivist culture of Asia and Latin America. Similar to our results, the study by Effron et al. (2018) also presents a reverse of the generally accepted proposition presented by authors like Doney et al. (1998) that “walk the talk” should be a more important component of trustworthiness in collectivist societies than in individualist societies. It has been observed that people from collectivist societies respond inconsistently across situations (Choi and Nisbett, 2000) and more specifically researchers like English and Chen (2007) have stated that Asians are expected to respond less consistently to hypocritic practices than western cultures. The same phenomenon is being observed in the study at hand as though the OH has a direct positive effect on JE; however in certain scenarios, if it leads to a considerable increase in PIB and PA, the total indirect effects become negative. Markus and Kitayama (1991) theory of independent and interdependent self-construal further explains the cross-situational inconsistencies among people. They are of the view that collectivist cultures foster an interdependent model of self which promotes connectedness. While the independent model of self-predominantly fostered in western cultures promotes a sense of autonomy and separation from others. The result of this study that OH has a positive effect on JE can be explained by the theory of the self as its theorists (for example, Suh, 2002; Cross et al., 2003; Church et al., 2014) also posit that among people who hold a more interdependent model of self, cross-situational inconsistency is less predictive of wellbeing. In these cultures even if inconsistent decisions lead to a lack of wellbeing, the arousal of cognitive dissonance (Heine and Lehman, 1997; Kitayama et al., 2004; Hoshino-Browne et al., 2005) is much lower than in western cultures (Savani et al., 2008). Finally and most importantly, one of the prime reasons for our study results is explained by Nisbett et al. (2001) stating that collectivist societies have a dialectical mode of reasoning in their everyday affairs. This form of reasoning along with the heightened interdependence embraces contradictions and paradoxes much more easily than in individualist societies that are more prone to an analytical model of reasoning (Peng and Nisbett, 1999).

The second reason for the results of this study was the prevalence of the COVID-19 pandemic during the data collection exercise. The COVID-19 pandemic has resulted in unthinkable death tolls throughout the world and dealing with the pandemic has presented challenges for nations and businesses alike. The literature is rife about the OH displayed by governments, institutions, and businesses in face of natural disasters (KyuJin and Yang, 2016). As mentioned, earlier organizations resort to OH and tactically employ inconsistencies in their talk, decisions, or actions in order to accomplish their goals in both normal business environments (Islam, 2018) and disaster situations (Wettenhall, 2014). COVID-19 is a historic natural disaster that has generated varied responses both positive and negative from organizations. Interestingly, there have been several studies on the hypocritic practices of the organizations during this pandemic, for example Ilsev and Aydin (2021) explored the emotional, attitudinal, and behavioral ramifications of leader hypocrisy for their organization and subordinates. Spicer (2020) focused on the effects of COVID-19 on Organizational culture and stated that the environmental jolts like the current pandemic makes the organizations hypocritical, that is, the organizations are compelled by the external environment to modify highly visible public aspects of their culture while leaving deeper assumptions and rituals intact. Similarly, Ashforth (2020) has evaluated the dynamics of identity and identification during and after the pandemic and states that a firm could portray itself as an “extended family,” but the hypocrisy could become immediately evident if the epidemic leads to a rapid release of personnel instead of seeking other methods to address the situation. These and many other studies have highlighted the constructive and disparaging responses of multiple organizations to this pandemic. The study that most closely relates to our study is by Gu et al. (2021). Although our study presents a positive relationship between OH and JE, the results from the Gu et al. (2021) state similar results by presenting an insignificant direct effect of hypocritically handled organizational response to COVID-19 pandemic on employee psychological withdrawal.

Lastly, Trocquenet-Lopez (2018) presents “hypocrisy as a necessary evil” or as Wingrove (2001) puts it: “to ask why hypocrisy is necessary is to ask why law, religion, honor, or public morality are altogether necessary” (p. 105). The necessity of hypocrisy has been emphasized by scholars especially in politics (McDonough, 2009; Tillyris, 2016; Grant, 2019), compromise (Kelly, 2021), creation and preservation of high morals (Taket, 1994; By and Burnes, 2013; Schwarze, 2020), goal accomplishment (Crouch, 2000), maintaining friendships (Grant, 2008), managing animates (Grant, 2008), meeting different and conflicting demands (La Cour and Kromann, 2011), relationships (Crouch, 2000), social life (Czarniawska, 2003; Jones, 2016; Albrecht, 2017), and upholding power (Eco, 2018) to mention a few with all having favorable outcomes for organizations. The necessity of hypocrisy has become a real issue for hospitality organizations also (Ari et al., 2020). The oxymoron of the hospitality sector is that, while trust and accountability are used to demonize OH, it is partly because of these values that drive OH to develop. Despite the fact that these organizations are believed to be capable of implementing transparent practices, the use of such rituals to structure power struggles ensures that OH will continue to be generated and help in reducing turnover and improving JE. The interest and aspirations of managers and frontline workers are conflicting and incompatible, thus, developing an organizational structure that is increasingly dependent upon OH. The display of the study result that OH is increasing the ability of an employee to stay embedded to the organization is partially due to the fact that OH has become a virtue in the Pakistani hospitality industry. It is the glue that holds together their virtuous aura, mask their inescapable vices, maintain organizational civility, reduce conflict and most importantly cultivate, support, and advance their preferable organizational policies.

Although the study presents a positive effect of OH on JE, the study also presents an underlying mechanism that ultimately makes this relationship negative through PIB and FA. As OH increases in the organizations, PIB between the organizational members also increases. The discrepancies between talk, decisions, and acts of not keeping the words to practice increase PIB between organizational members. These inconsistencies in words and their respective actions affect the behaviors of employees and their ability to believe their supervisors, peers, and subordinates. Thus, the prevalence of OH in hospitality organizations leads to PIB, which in these institutions is a lack of conviction that others in the group could be relied upon (Bashir and Nasir, 2013), could act in accordance with their statements and promises (Said et al., 2021), are honest in their negotiations and are not exceedingly opportunistic in taking advantages (Karatepe et al., 2021). In summary, the inconsistencies of deeds and insincerity of words if reflected in the hospitality organizations decrease belief in other individuals and especially in the words and deeds of their leaders. It is also reported in this study that although OH leads to an increasing feeling of PIB in frontline employees, however, it does not always lead to a decrease in the ability of employees to display JE contrary to study hypothesis that PIB will mediate the relationship between OH and JE. The study by Wu et al. (2021) most accurately support our results using the research context of the COVID-19 pandemic to evaluate various forms of PCBs in a hospitality organization. The results of their study provide similar results as they conclude that not all PCB leads to unfavorable outcomes. In a crisis, FA and lack of JE originate if managers and frontline workers do act with mutual consideration and do not fulfill corresponding obligations.

OH is significantly related to FA as it stems from the belief that there is a lack of integrity in the organizations, and it is acting with dual standards (Baumgartner et al., 2008). The study results conclude that inconsistencies and disconnection between the words and deeds lead to negative attitudes like FA. Furthermore, while acting as a mediator, it results in organizational inefficiencies and lack of JE (TellIoglu, 2021) and providing support for the study hypothesis.

This study ultimately hypothesized that OH through PIB and JE declines an employee's ability to display JE which have been proven by the study results. It occurs when hospitality institutions strive to meet the needs of frontline workers and confronted with a plethora of competing ideas and demands, widening the gap between reality and desired self-descriptions. These expectations, diverse values, and environmental pressures lead to FA (Kandasamy and Ancheri, 2009). According to Wu et al. (2021) in hotel organizations, negative outcomes like FA only exist if both the managers and frontline workers do not operate with mutual consideration and do not fulfill corresponding obligations. Similarly, if members of an organization think that their leaders do not live up to their members' expectations in terms of justice, they may conclude that their leaders do not walk the walk. Finally, in the hospitality industry during this pandemic crisis situation, the onus falls both on the managers as well as frontline workers; if they work with mutual consideration, the sense of PBCs would be limited. Both the parties may not be able fulfill all their committed obligations during the normal circumstances however keeping in view the COVID-19 crisis situation they may cut each other some slack by diminishing FA and maintaining JE.

### Theoretical implications

First, this study extends the existing literature on OH by discussing the underlying mechanisms related to internal stakeholders. Prior studies predominantly focus on employees' perception of OH. This study focused on how and if it has detrimental effects in a situation-specific scenarios. Specifically, keeping in consideration the current COVID-19 pandemic, a positive effect of OH on JE was reported contrary to the existing knowledge. However, there is adequate support for the positive results in national culture (English and Chen, 2007), crisis management (Wettenhall, 2014), and hypocrisy literature (Wingrove, 2001). A supplementary data analysis reported a nonlinear relationship between OH and JE providing support to both lines of the argument, thus, it is recommended for future studies to focus on curvilinear relationships between these variables in situation-specific investigations. Second, by evaluating the direct and indirect effects of OH, this study presents a more precise understanding regarding its effects on employee outcomes and adds empirical evidence to otherwise exploratory literature. Third, the literature on OH is primarily focused on its antecedents (Lee et al., 2016) and this study contributes novel insights into its outcomes. This research presents an underlying mechanism that on the surface display, positive outcomes of OH, however, indirectly lead to negative psychological (increased PIB), emotional (increased FA), and behavioral (decreased JE) reactions, thus, fulfilling a research gap. The existing research on OH focused on its negative outcomes; this study, however, investigated its holistic effects on the perceptions, emotions, and behaviors of frontline employees.

### Managerial implications

This study has important practical implications. First, although on the face, OH may have favorable outcomes, but ultimately it harms the psychological, emotional, and behavioral outlook of frontline workers. Organizations make considerable efforts to maintain their legitimacy, authenticity, credibility, and ethical image in the external environment. Similar efforts may be initiated for internal stakeholders to reduce their feelings of OH. Second, the overall model of the study shows that OH has a negative indirect effect on JE and also reveals that OH generates negative outcomes like PIB and FA which in turn reduce their ability to stay embedded in the organization. As employees are the core resource of any organization, they must take initiatives to limit any OH practices, ensure that they walk the talk so that employees come across as few hypocritical actions as possible, and improve JE. Third, this study also indicated that with an increased display of OH, employees experienced a strong feeling of PIB as well as anxiety in the form of FA.

An elevated feeling of OH raises questions about the organization's real motives and moralities in the minds of its members and their overall attitude toward the organization is adversely impacted. These findings have important implications for organizations in general and hospitality organizations in specific as employees have insider information about their real motives and morality, which could have grave consequences for their overall business performance. Especially in the age of social media and digital word of mouth, employees' lack of confidence in their institution due to OH could reach the wider world which in the hospitality industry could be detrimental. The hospitality industry needs to pay attention toward their employees' perceptions about OH and take initiative to reduce these harmful perceptions, thus, rescuing their at stake reputation. Lastly, and most importantly, this study presents that OH could make the employees perceive that the inducements committed to them have not been fulfilled, which generates anxiety about their future which in turn leads to lower embeddedness toward their jobs. Given that in a service industry like hospitality, employees are an irreplaceable resource to an organization that could provide you with competitive advantages, hotel organizations need to align their values with that of their employees by word and actions or risk losing them.

### Limitations and future research

This study has some limitations that also provide opportunities for future research. First, this study is only focused on frontline employees from the hospitality industry and does not include employees from other sectors like manufacturing, supply chain, or marketing. The experiences of employees other than frontline employees may be different and may perceive OH differently. Thus, for future studies, the study participant should be from other sectors and industries to improve the generalization of results. Second, as this study is conducted in an Asian context with its participants from Pakistan, the external validity of its results may be limited. The perception of the employees may vary across the globe; so future studies should focus on other nationalities and cultures. Third, the data were collected during the COVID-19 pandemic, the world economy was in complete recession, and the unemployment rate was at an all-time high in the majority of countries. The situation was much more severe for the hospitality industry as the stingiest restriction has been applied to them. The cruise industry was almost eliminated, airlines could not fly customers, and cross border and internal tourist restrictions have existential challenges for the hospitality industry. As employees did not have job opportunities available even if they perceived that their organization displays OH, it is essential to understand how these employees would react when there are adequate job opportunities. It is proposed that the effects of construct-related national culture, other crisis situation, and prevalence of hypocrisy as a necessary evil should be assessed for future studies. Additionally future studies should assess and validate the curvilinear relationships identified in the supplementary analyses section of this investigation.

## Conclusion

In conclusion, this study contributes significantly to the JE research. This study provides empirical evidence as how industry and environment-specific variable increase or decrease the ability of an employee to stay embedded in the organization. Specifically, OH is certain environment increasing this ability, while in the same environment through complex interventions like PIB and FA, it ultimately decreases this ability. This conceptual model and results of this study provide a theoretical foundation to guide and enhance future research as well as help practitioners find ways and conditions to retain valuable employees.

## Data availability statement

The raw data supporting the conclusions of this article will be made available by the authors, without undue reservation.

## Ethics statement

This study was approved by the Institutional Review Board (IRB) of Qingdao University. The synopsis of the study was presented by the researchers to the IRB, which was duly approved based on ethical considerations. Furthermore, the IRB also ensured that the study is performed in accordance with the Declaration of Helsinki.

## Author contributions

All authors developed the study aims, analyzed the papers referenced within, and actively participated in developing the critiques, theories, and conclusions expressed within the paper. All authors contributed to the article and approved the submitted version.
